# Ganoderic Acid in the Treatment of Prostate Cancer

**Published:** 2012-08-25

**Authors:** Abdulghani Ameri

**Affiliations:** 1 Department of Drug and Food Control, School of Pharmacy, Ahvaz Jundishapur University of Medical Sciences, Ahvaz, IR Iran

**Keywords:** Ganoderic Acid, Prostate Cancer

Prostate cancer is the most commonly diagnosed cancer in men and is the second most common cause of cancer-related deaths in the western world. Presently, the most common options for treatment are prostatectomy, radiation and chemotherapy. While certain immunotherapeutics have shown promise in clinical trials, the search for more effective, long-term treatment of later stages continues (1). Ganoderma lucidum is a species of the Basidiomycetes that is widely used in Traditional Chinese Medicine for prevention and treatment of a variety of human ailments including hypertension, bronchitis, arthritis, neurasthenia, hepatopathy, chronic hepatitis, nephritis, gastric ulcer, tumorigenic diseases, hypercholesterolemia, immunological disorders and scleroderma (2). Antitumor and immune enhancing properties of G. lucidum with no cytotoxicity makes it a good candidate to be effective in preventive oxidative damage and resulting disease (3).

Ganoderic acids (GAs) are a class of closely related triterpenoids (derivatives from lanosterol) found in Ganoderma mushrooms. There have been efforts to identify the chemical constituents that may be responsible for the putative pharmacological effects. There are dozens of ganoderic acids that have been isolated and characterized, of which ganoderic acid A and ganoderic acid B are the best characterized. Some ganoderic acids have been found to possess biological activities including hepatoprotection, anti-tumor effects and 5-alpha reductase inhibition (4).

One type of Ganoderic Acid (GD-DM), induces toxicity in both androgen dependent and independent prostate cancer. Prostate cancer cell lines (PC-3 (PC cell lines have high potential for metastasis) and LnCaP) were shown to be inhibited by GA-DM treatment in a dose dependent manner (5). Although there is no documented data involving the use of GA-DM in primary prostate tumor cells, the effectiveness of the drug in these cell lines suggests that GA-DM might work in attenuating the growth of primary tumors (5). LnCaP cells have been shown to retain androgen dependence and maintain the presence of androgen receptor (AR), thus resembling early-stage prostate cancer. PC-3 cells are androgen-independent and express little to no prostate specific antigen (PSA), two common features of metastatic, later-stage prostate cancer (5).

It has also been shown that GA-DM treatment inhibits both the activity of 5- α-reductase and the conversion of testosterone to dihydrotestosterone (DHT). The inhibition of DHT activity possibly occurs due to the conformational similarity in the four-ringed steroidal structures of DHT and GA-DM. GA-DM competitively blocks the AR, preventing DHT binding and obstructing the normal DHT-mediated signaling pathway (5). While the effect of GA-DM on both 5-α-reductase and the AR mirrors that of other steroidal inhibitors, an even more promising effect of GA-DM is its inhibition of osteoclastogenesis. Osteoclastogenesis is a significant factor in prostate cancer metastasis, and GA-DM has been shown to limit this process in a pre-osteoclastic cell line (5). Several studies indicate that GA-DM treatment increases bone density and decreases osteoclastogenesis, suggesting that GA-DM could be a potential therapeutic alternative in prostate cancer. GA-DM can act through down-regulation of C-Fos (a protein encoded by the FOS gene) and NFATc1 (nuclear factor of activated T-cells, cytoplasmic 1 is a protein that in humans is encoded by the NFATC1 gene), which also regulate osteoclastogenesis as a result of stimulation through RANKL (receptor activator of nuclear factor kappa- B ligand) (6).
